# *Alaria alata* Mesocercariae among Feral Cats and Badgers, Denmark

**DOI:** 10.3201/eid2110.141817

**Published:** 2015-10

**Authors:** Nao Takeuchi-Storm, Mohammed N.S. Al-Sabi, Stig M. Thamsborg, Heidi L. Enemark

**Affiliations:** Technical University of Denmark, Copenhagen, Denmark (N. Takeuchi-Storm, M.N. Solaiman Al-Sabi, H.L. Enemark);; University of Copenhagen (S.M. Thamsborg)

**Keywords:** *Alaria alata*, Mesocercariae, zoonoses, Alariosis, parasites, trematode, cat, badger, domestic pig, wild boar, cox1, Denmark

**To the Editor:** The digenean trematode *Alaria alata* is considered an emerging zoonotic parasite in Europe because of increased findings in wild boars during *Trichinella* inspection. No human illness caused by *A. alata* mesocercariae (infective larvae) has been reported, but concern remains because the closely related North American species *A. americana* has caused illnesses among humans, including 1 death (*1*).

In Denmark, high prevalence of *A. alata* trematodes in final hosts has been shown (*2*), but limited data on potential paratenic hosts are available. Therefore, samples from 406 domestic pigs, 130 wild boars, 9 badgers, and 99 cats were collected by convenience sampling during October 2013–September 2014. We used pig and wild boar samples from multiple geographic areas of Denmark were leftover tissue samples from ongoing *Trichinella* spp. surveillance. Badgers had died naturally or were hit by vehicles (8 from Jutland, 1 from Zealand) and collected as part of a wildlife monitoring program. Cats (all from Zealand) were either feral (n = 92) or domesticated (n = 7) and had been euthanized as part of a national control program. Carcasses were necropsied in our laboratory; we collected 30 g of tissue samples according to Riehn et al. (*3*). All samples were analyzed by the modified *A. alata* mesocercariae migration technique (*3*). In brief, the sample was cut into ≈0.5-cm edge pieces, wrapped in gauze, and suspended for 90 min by 2 wooden sticks in a conical glass with ≈300 mL of water (46°C–48°C). Approximately 15 mL of sediment was collected from the bottom of the glass by suction by using a glass pipette and examined by microscopy (magnification ×20).

*A. alata* mesocercariae were isolated from 3 cats and 6 badgers (Technical Appendix Table 1). All 3 cats were female (2 pregnant, 1 lactating); prevalence was significantly higher in pregnant or lactating females (3/12) than other intact females (0/24) (p = 0.031 by Fisher exact test). This finding might be related to increased exposure because an increase in predation by the cats during pregnancy and lactation to meet higher protein and energy demand. However, because *A. marcianae* mesocercariae can be transmitted through milk in cats (*4*), lactating females may also be predisposed to an increased chance for *A. alata* mesocercariae reaching their offspring. Examination of the intestines of all cats by sedimentation and counting technique (*5*) revealed no *A. alata* adults. Although *A. alata* adults have been found in cats in Uruguay (*6*), reports from Europe are lacking, and thus it is still uncertain whether cats can act as amphiparatenic or final hosts. Natural infection of cats with other *Alaria* spp. has been reported in the United States (*7*), indicating biologic differences among *Alaria* spp.

Zoonotic risk for *A. alata* infection through ingestion of cat meat is probably minimal in Europe but may be important in Asia and South America, where cats are occasionally consumed. Badgers are, however, sometimes consumed as game meat or road kill meat in Europe. In Russia, 10.6% of trichinellosis outbreaks during 1998–2002 were caused by consumption of badger meat (*8*). Thus, the zoonotic potential of infections in this animal, although a protected species, should not be ignored. 

Negative findings in domestic pigs and wild boars in this study may reflect underestimation because those samples were below the recommended 30 g and often taken from sites that are not typically infected with mesocercariae (*3*). A follow-up study with better sampling strategy would be of value to determine the risk for *A. alata* transmission from domestic pigs and wild boars.

Identification of isolated mesocercariae was confirmed by PCR and sequencing of a fragment (332 bp) of the mitochondrial cytochrome c oxidase subunit 1 gene (*cox*1) (*9*). By neighbor-joining analysis (*10*), the consensus *cox*1 sequences were compared with the trematode *Neodiplostomum seoulense* (outgroup), 1 *A. alata* isolate from a Danish red fox, and all 7 *cox*1 sequences of *Alaria* spp. available in GenBank as of October 2014. (Sequences from this study have been deposited into GenBank under accession nos. KP123417–KP123422 [badgers] KP123423–KP123425 [feral cats].) The inferred phylogenetic tree (Figure) showed marked genetic variation among *A. alata* isolates from Denmark and other parts of Europe but no apparent separation of most *A. alata* isolates from Europe based on host species or country, except for that from badger 1 (Technical Appendix Table 2). This animal originated from Northern Jutland, where host and parasite populations are geographically isolated by a large fjord separating the region from the rest of the country. The marked genetic variation within *cox*1 sequences suggests the usefulness of this marker, but additional genetic markers should be included in future studies to explore the genetic flow of *A. alata* within natural hosts.

**Figure:**
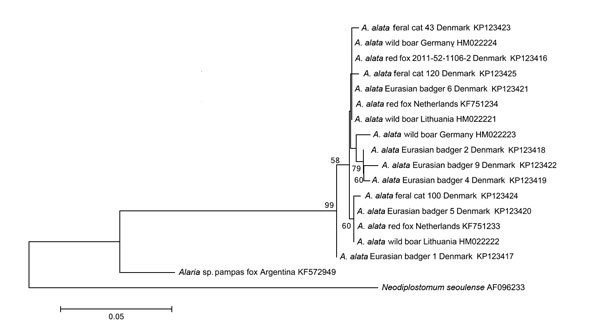
Neighbor-joining phylogenetic tree of *Alaria* *alata* isolates based on the analysis of partial mitochondrial cytochrome c oxidase subunit 1 gene sequences (332 bp). Bootstrap values are indicated to the left of the nodes and are based on 10,000 replicates. GenBank accession numbers are listed to the right. Scale bar indicates base substitutions per site.

In conclusion, *A. alata* mesocercariae seem to favorably infect pregnant or lactating cats, thereby increasing the chance of vertical transmission. Further, detection of *A. alata* infection in numerous badgers suggests potential high zoonotic risk associated with ingestion of such exotic meat. These results should, however, be interpreted with caution because of the small sample size and unknown efficacy of the modified *A. alata* mesocercariae migration technique.

Technical Appendix Detailed information about origin and type of samples as well as characteristics of *A*. *alata* mesocercariae–positive animals.
