# Supplementary material for: Alaria alata Mesocercariae among Feral Cats and Badgers, Denmark
Source: Emerg Infect Dis. 2015 Oct;21(10):1872–4. doi: 10.3201/eid2110.141817 (PMC4597358; doi:10.3201/eid2110.141817)
Supplement: Technical Appendix — Detailed information about origin and type of samples as well as characteristics of A. alata mesocercariae–positive animals. [file 14-1817-Techapp-s1.pdf]

Article DOI: http://dx.doi.org/10.3201/eid2010.141817

Alaria alata Mesocercariae among Feral Cats and Badgers, Denmark

Technical Appendix

Technical Appendix Table 1. Information on domestic animals and wildlife examined for *Alaria alata* by using modified *Alaria alata* migration technique, Denmark October 2013–September 2014*

Host species	Origin	Examination period	Sample type	No. examined	Prevalence (%) (95% CI)
Pigs					
Outdoor	Z	Oct 2013–Mar 2014	Diaphragm or foreleg	260	0
ND	Z, F, J, ND	Oct 2013–Mar 2014	Diaphragm or foreleg	146	0
Wild boars					
Farmed	Z	Oct 2013–Mar 2014	Diaphragm or foreleg	39	0
Farmed	J	Nov 2013–Jan 2014	Diaphragm, foreleg, or intercostal muscles	58	0
ND	ND	Oct 2013 – Mar 2014	Diaphragm or foreleg	33	0
Badgers	J	Feb–Sep 2014	Tissue sample mixture†	9	66.7 (29.9–92.5)
Cats	Z	Mar–May 2014	Tissue sample mixture†	99	3.0 (0.6–8.6)

*Z, Zealand; F, Funen; J, Jutland; ND, no data available.
†30 g tissue sample mixture of diaphragm, tongue, and masticatory muscles;

Technical Appendix Table 2. Characteristics of *Alaria alata* mesocercariae–positive animal tissue samples identified by using modified *Alaria alata* migration technique, Denmark

Identity code	Species	Sex	Origin	No. of mesocercariae per 30 g sample	GenBank accession number
Cat 43	Cat (feral)	Female (pregnant)	Zealand	23	KP123423
Cat 100	Cat (feral)	Female (pregnant)	Zealand	2	KP123424
Cat 120	Cat (feral)	Female (lactating)	Zealand	12	KP123425
Badger 1	Badger	Not recorded	Northern Jutland	1*	KP123417
Badger 2	Badger	Female	Central Jutland	2	KP123418
Badger 4	Badger	Female	Southern Jutland	1	KP123419
Badger 5	Badger	Male	Central Jutland	2	KP123420
Badger 6	Badger	Male	Central Jutland	8	KP123421
Badger 9	Badger	Female	Zealand	2	KP123422

*30 g tissue sample mixture of diaphragm, tongue, and masticatory muscles were used.
